# Antioxidant: Antimycobacterial and Antibiofilm Activities of Acetone Extract and Subfraction *Artemisia afra* Jacq. ex Willd. Against *Mycobacterium smegmatis*

**DOI:** 10.3390/antibiotics13111027

**Published:** 2024-10-31

**Authors:** Mabasa Precious Matlala, Mashilo Mash Matotoka, Wanda Shekwa, Peter Masoko

**Affiliations:** Department of Biochemistry, Microbiology and Biotechnology, University of Limpopo, Private Bag X1106, Sovena 0727, South Africa; matlalaprecious2015@gmail.com (M.P.M.); mashilo.matotoka@ul.ac.za (M.M.M.); wanda.shekwa@ul.ac.za (W.S.)

**Keywords:** medicinal plants, antioxidant, *Mycobacterium smegmatis*, anti-biofilm formation

## Abstract

Tuberculosis is a worldwide prevalent and recurring disease that contributes significantly to high mortality rates. This study aimed to investigate the antioxidant, anti-mycobacterial, and antibiofilm activities of *Artemisia afra* acetone crude extract. **Methodology:** The crude acetone extract was fractionated using column chromatography and characterized by liquid chromatography–mass spectroscopy (LC-MS). A 2,2-diphenyl-1-picrylhydrazyl (DPPH) free radical scavenging assay was used to assess the antioxidant activity. The antimycobacterial activity against *Mycobacterium smegmatis* was screened using bioautography, broth microdilution, and growth curve assays. Molecular docking was used to predict the possible mechanisms of action of the LC-MS-identified ligands. Crystal violet was used to screen for anti-cell adherence and biofilm inhibition activities. **Results:** The crude extract scavenged 77% of the free radical at 16 μg/mL. The subfraction had a lower minimum inhibitory concentration (MIC) (0.078 mg/mL) compared to the crude extract (0.313–0.833 mg/mL). The subfraction had a concentration-dependent inhibition effect (>50%) on mycobacterial cell adherence and early biofilms. However, the mature biofilms were resistant. Two propanoate compounds, [(2S)-3-[6-acetyl-4,6-dihydroxy-3-[(1R)-1-hydroxyethyl]tetrahydropyran-2-yl]-2-hydroxy-propyl] (2R)-2-amino-3-(1H-imidazol-5-yl)propanoate and 3-(6-aminopurin-9-yl)propyl 3-(2,4-dioxo-1,3-diazaspiro[4.5]decan-3-yl) propanoate, had binding energies of −5.4 kcal/mol and −6.3 kcal/mol, respectively, against the RNA polymerase binding protein. **Conclusions:** The results show that *A. afra* acetone crude extract has antioxidant and antimycobacterial activities that can be improved by fractionation.

## 1. Introduction

Tuberculosis (TB) has been known for decades as the predominant communicable disease that contributes greatly to morbidity and mortality rates around the world, with an increasing number of drug-resistant strains [1]. Consequently, it is also one of the top ten deadly diseases in South Africa and around the world [2]. The main pathogen of TB is *Mycobacterium tuberculosis*, which mainly infects the lungs, although it can invade other organs in a host, such as the kidneys, spine, bones, etc. [3]. The spread of the disease is primarily through aerosols released in coughs and sneezes from infected individuals [4]. The World Health Organization (WHO) has previously reported that around 10 million people worldwide were infected and approximately 1.3 million mortalities were recorded in the year 2020 [5].

Treatment regimens for TB consist of a combination of antibiotics, such as isoniazid (INH), ethambutol (EMB), pyrazinamide (PZA), and rifampicin (RIF). The emergence of multidrug-resistant and extensive-resistant TB strains has led to a reduction in the efficacy of the regimen [5,6,7]. Screening for new drugs using *M. tuberculosis* has been challenging due to its virulence; therefore, microbes from the same genus, such as *Mycobacterium smegmatis*, are commonly used in preliminary investigations [8,9]. *M. smegmatis* is a fast-growing saprophytic microorganism closely related to *M. tuberculosis* in that they share genes that allow them to adapt to stressful conditions [10]. *M. smegmatis* and *M. tuberculosis* are similar in cell wall structure and biochemical properties. The cell walls of *M. tuberculosis* and *M. smegmatis* consist of mycolic acids that contribute to their resistance to drugs by blocking the entry of hydrophobic and hydrophilic molecules [11,12]. As a result, *M. smegmatis* serves as a common surrogate for screening for antimycobacterial potential.

Medicinal plants produce bioactive compounds that possess anti-inflammatory, anticancer, antioxidative, and antimicrobial activities to mention but a few [13,14]. The use of numerous plants to treat TB and associated symptoms such as fever, chest pains, and coughs has previously been reported [15]. *Artemisia afra* Jacq. ex Willd. (African wormwood) is an evergreen plant from the Asteraceae family. It produces small yellow flowers and grows from 0.6 to 2 m tall [16]. This plant has a wide distribution in Zimbabwe, South Africa, Ethiopia, Lesotho, Namibia, and Swaziland, where it is traditionally used for the treatment of various diseases, such as asthma, cough, influenza, and diabetes [17]. In South Africa, it is locally known as Umhlonyane (isizulu), African wormwood (English), Lanyana (Sotho), and Lengana (Tswana) [18,19]. Flavonoids, terpenes, sterols, terpenes, and acetylenes have been isolated from several plants belonging to the Asteraceae family, including *A. afra* [20]. *A. afra* has been recognized for its extensive antimicrobial properties, targeting a broad range of microorganisms, such as *Actinomyces israelii,* methicillin-resistant *Staphylococcus aureus, Streptococcus mutans*, *Prevotella intermedia, Porphyromonas gingivalis*, and *C. albicans* [21,22,23]. These findings, together with the ethnobotanical use of *A. afra* leaves, suggest that it possesses phytochemicals that hold promising potential as alternatives to conventional antibiotics. This study aimed to investigate the antioxidant, antimycobacterial, and antibiofilm activities of the crude acetone leaf extract and fractions of *Artemisia afra* and to use molecular docking to assess possible the molecular interactions of the identified phyto-compounds during antimycobacterial action. Furthermore, the antibiofilm activity of the extracts was evaluated by determining the anti-cell adherence and early and mature biofilm eradication activities.

## 2. Results and Discussion

### 2.1. Phytochemical Analysis

The total phenolic content in the crude extracts was found to be 599.7 ± 3.5 mg GAE/g, the total flavonoid content was 60.6 ± 1.3 mg QE/g, and the total tannin content was 87.2 ± 4.1 mg TAE/g (Table 1). Flavonoids have demonstrated exceptional ability in reducing ROS production and possess metal-chelating properties [24]. Tannins are recognized for their antibacterial effects, which they achieve by interacting with cell membranes or forming metal complexes [25]. The high content of polyphenolic compounds in *A. afra* underpins the plant’s biological activity.

### 2.2. Antioxidant Activity

**Figure 1:**
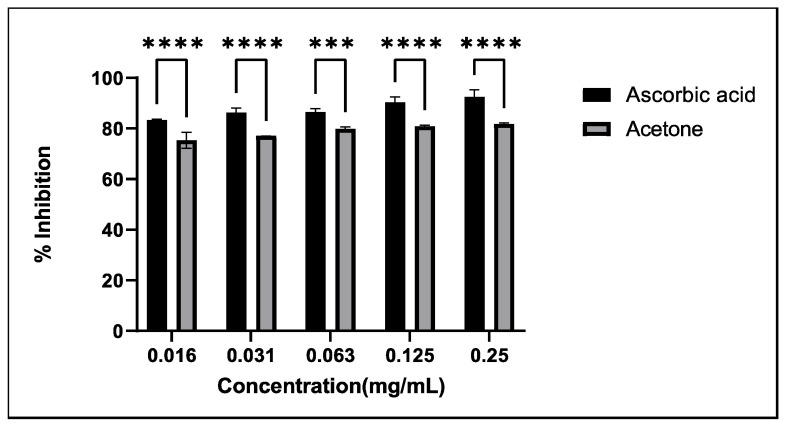
Percentage of free radical scavenging activity of *Artemisia afra* acetone extract. L-ascorbic acid was used as a positive control. The error bars on the graphs indicate the standard deviation from the mean (*n* = 3). Dunnet’s multiple comparison test was used to compare the columns using graph pad prism 9. (****): *p* ˂ 0.0001; (***): *p* ˂ 0.001.

Active tuberculosis has been associated with the initiation of oxidative stress, which can lead to immunosuppression by weakening T-cell-mediated immunity. Oxidative stress also promotes the proliferation of Mycobacteria [26]. The antioxidant potential of *A. afra* acetone extract was assessed using DPPH as a free radical, with ascorbic acid serving as the positive control (Figure 1). The findings reveal that the crude extract inhibited over 75% of the free radical activity, comparable to that of ascorbic acid. Three compounds isolated from *A. afra*—scopoletin, acacetin, and betulinic acid—exhibited significant antioxidant activity, with scopoletin achieving an IC_50_ value of 1.24 μg/mL, which was nearly identical to that of vitamin C (1.22 μg/mL) [27]. In vivo studies suggested that managing oxidative stress through antioxidant supplementation is beneficial for preventing and treating TB infections [28]. Additionally, exogenous antioxidants may help mitigate anti-TB drug-induced toxicity [29].

### 2.3. Cytotoxicity

Alveolar macrophages play a key role in the innate immune response and are essential during Mycobacterial infection, making it important to evaluate the cytotoxic effects of plant extracts on macrophage cell lines. The crude acetone extract showed no cytotoxicity toward THP-1 macrophages, with an LC_50_ value of 172.7 µg/mL determined (Table 1). Testing plant extracts on THP-1 macrophages assist in assessing the direct cytotoxic effects on immune-like cells, predicting their potential toxicity in humans, particularly in the context of immune response modulation. It is generally accepted that LC_50_ concentrations below 20 μg/mL are considered toxic, and those above 20 μg/mL are non-toxic [30,31]. In a different study, *the A. afra* extract tested did not induce cell cycle arrest and was not cytotoxic to eukaryotic cells [32]. Furthermore, *A. afra* extracts were shown to have a low cytotoxic effect against Vero cells with an LC_50_ value ˃ 500 µg/mL [17]. These studies suggest safety in the use of *A. afra* extracts. Rifampicin was found to be non-toxic (CC_50_, 520.02 μg/mL) when tested against mammalian Vero E6 cells [33]. Evaluating the combinational effects of first-line drugs (rifampicin and isoniazid) with *A. afra* extracts is necessary to explore the potential of the phytochemicals as adjuncts to chemotherapy. 

### 2.4. Antimycobacterial Activity

The minimum inhibitory concentrations (MICs) of the crude extracts and fractions against M. smegmatis planktonic cells were assessed. The crude acetone extracts exhibited an average MIC value of 0.5 ± 0.22 mg/mL (Table 1). In a separate study by Masoko and Nxumalo [34], the acetone leaf extract of *A. afra* showed antimycobacterial activity with an MIC of 0.39 mg/mL against *M. smegmatis*. Additionally, the ethanol leaf extract of *A. afra* was reported to have an MIC of 1.56 mg/mL against *M. smegmatis* [35]. The dichloromethane extract of *A. afra* demonstrated bactericidal activity against the *M. smegmatis* mc26230 strain under hypoxic conditions and in the presence of various carbon sources [36,37]. This study validates the antimycobacterial activity of crude acetone extracts of *A. afra*, prompting their fractionation. 

### 2.5. Bioassay-Guided Fractionation

Crude extracts from plants can contain a vast array of compounds, sometimes numbering in the hundreds or thousands. By fractionating the extract, the mixture is simplified, making it easier to analyze, investigate, and comprehend the characteristics of each component [38]. The crude acetone extract underwent fractionation via column chromatography. The extracts were combined due to their comparable phytochemical profiles as determined by thin-layer chromatography. The minimum inhibitory concentration (MIC) for fraction F_C2_ was found to be 0.078 mg/mL, which is significantly lower than that of the crude extract at 0.52 ± 0.22 mg/mL and the first column fraction (F_C1_) at 0.90 ± 0.10 mg/mL (Table 1). These findings indicate that further fractionation of the crude extract and FC1 into F_C2_ enhanced the antimycobacterial activity. Muleya et al. [39] reported that fractionation improved activity after conducting a study on *A. afra* and found that the methanol and acetone fractions had good activity against Escherichia coli. Therefore, when working with *A. afra*, further purification of its crude extracts is essential to enhance their antimycobacterial activity. Isoniazid and rifampicin have been reported to have MIC values ranging from 1 μg/mL to 4 μg/mL against MDR TB isolates [40,41]. Biological systems are intricate, and diseases typically affect various targets or pathways. Extracts that are fractionated and contain multiple compounds may exhibit a wider range of activity, potentially enhancing their effectiveness against mycobacterial infections [42]. Consequently, further fractionation was halted to allow for a more in-depth assessment of the antimycobacterial activity of F_C2_.

### 2.6. Growth Curves

**Figure 2:**
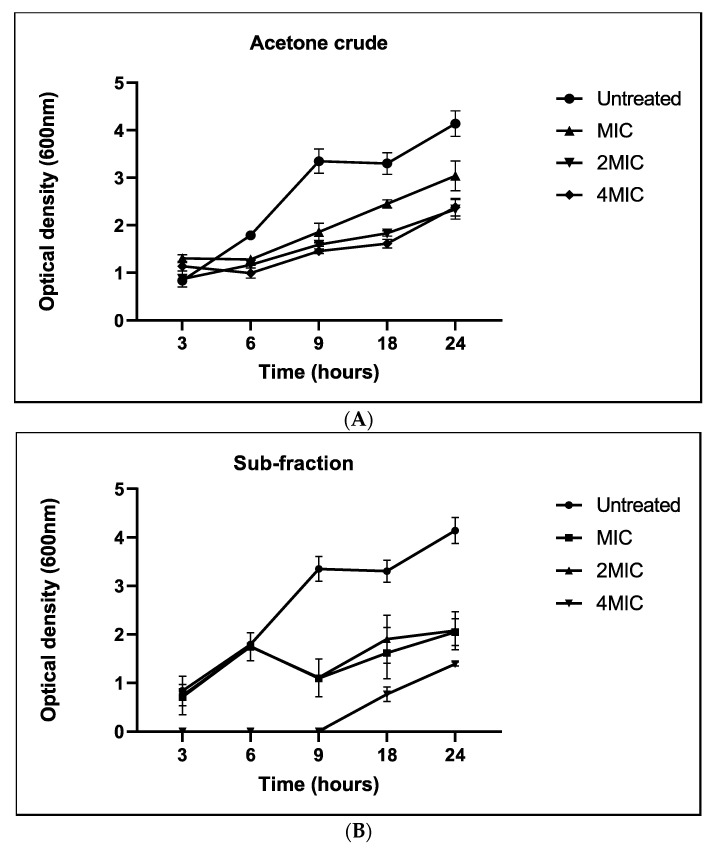
Growth curves of *M. smegmatis* planktonic cells upon treatment with acetone crude extract (**A**) and the subfraction (**B**) monitored at different time intervals and measured at 600 nm. The error bars on the graphs indicate the standard deviation from the mean (*n* = 3).

A growth curve assay was used to monitor the growth of *M. smegmatis* planktonic cells after treatment with the crude extract and (F_C2_). The crude extract was able to inhibit the growth of *M. smegmatis* cells in a concentration-dependent and bacteriostatic manner for 24 h (Figure 2A). At 4×MIC (0.312 mg/mL) (F_C2_), the growth of the culture was completely arrested for approximately 9 h before the culture recovered (Figure 2B). Our findings support the hypothesis derived from experiments indicating that bacteria develop longer lag times when exposed to antibiotics, coinciding with the introduction of new nutrients [43,44]. Polyphenols, flavonoids, and tannins have been reported to disrupt microbial growth by inhibiting efflux pumps interfering with cell membrane permeability and integrity, among other functions [45]. Indeed, one of the first-line drugs, isoniazid (INH), inhibits bacterial cell wall synthesis [46]. 

### 2.7. Antibiofilm Activity

**Figure 3:**
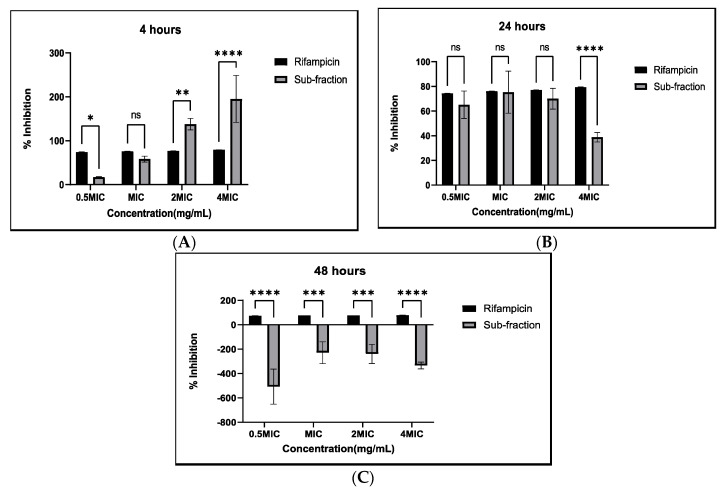
Antibiofilm activity of *Artemisia afra* plant extracts measured at different time intervals during initial cell attachment (**A**), early preformed biofilm biomass (**B**), and mature biofilm biomass (**C**). Percentage inhibition ≥ 100%: complete eradication ≥ 50%: good inhibition; ˂50%: poor inhibition; ˂0%: enhanced biofilm formation. Rifampicin served as a positive control. The error bars on the graphs indicate the standard deviation from the mean (*n* = 3). Dunnet’s multiple comparison test was used to compare the columns using GraphPad Prism 9. ns: not significant. (*): *p* < 0.05. (**): *p* < 0.01. (***): *p* < 0.001. (****): *p* < 0.0001.

**Figure 4:**
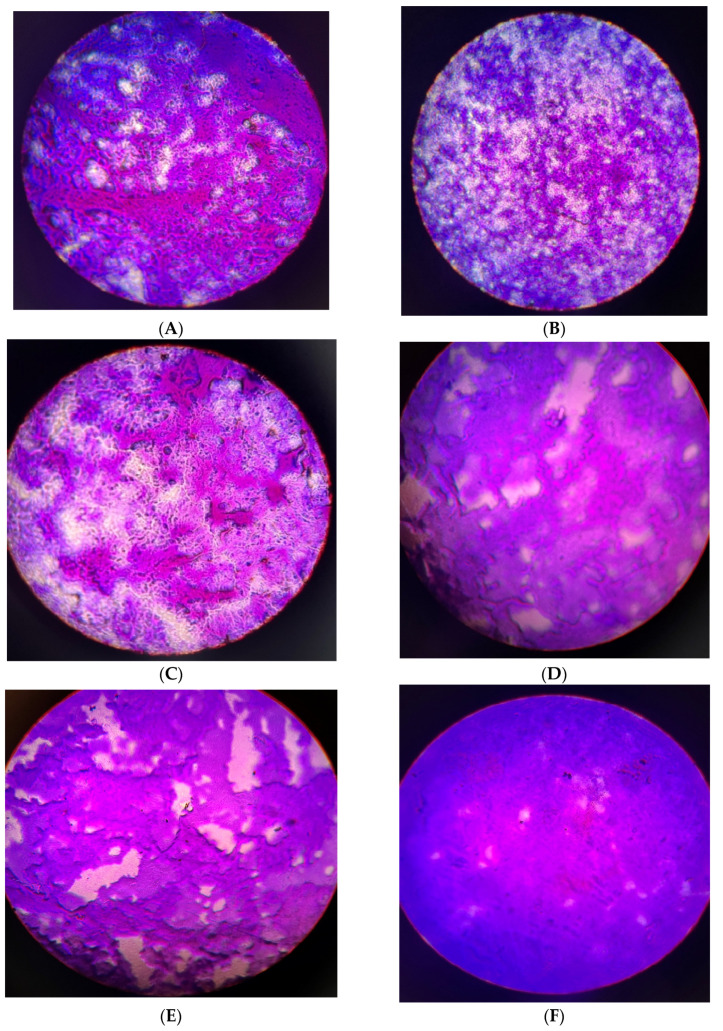
Light microscopic images (40X magnification) of crystal violet-stained early pre-formed biofilms grown on cover slips submerged in growth media. The preformed biofilms were treated with 4×MIC (**A**), 2×MIC (**B**), MIC (**C**), 0.5×MIC (**D**) of extract, and rifampicin (1 μg/mL) (**E**). An untreated biofilm was used as a negative control (**F**).

Planktonic cells of Mycobacteria develop biofilms as a virulence factor to enhance their pathogenicity and resilience in challenging environmental conditions. The integrity of a biofilm primarily relies on the extracellular polymeric substances (EPS) and the metabolic activities of the biomass within the biofilm [47]. Thus, it is essential to explore the effectiveness of crude extracts and their fractions in preventing biofilm formation and eliminating preformed biofilms created by microbes. In this study, it was noted that the initial phase of cell attachment of planktonic cells was more vulnerable to treatment with subfractions (Figure 3A). The activity might be due to interference with hydrogen bond formation, electrostatic, and van der Waals forces because these factors are important in cell adhesion [48,49]. Eradicating preformed biofilm biomass proved more difficult, with the highest concentration showing decreased inhibitory activity (Figure 3B and Figure 4A). Micronutrients, sugars, and polyphenolics present in plant extracts can potentially promote biofilm formation by providing essential nutrients and signaling molecules that support the growth and structural development of biofilms. Furthermore, it was discovered that subfraction (F_C2_) enhanced the formation of biomass of a mature 48 h biofilm, and similarly, rifampicin significantly lost efficacy (Figure 3C). Cell wall-targeting antibiotics trigger lag-phase bacteria to generate surface-mediated filaments, which enhance the formation of biofilms and aggregates [50]. Medicinal plant extracts have been shown to enhance the growth of microorganisms [51], which could be the effect of the increased biofilm biomass observed after 48 h of treatment. On the other hand, Priyanto et al. [52] reported the efficacy of crude fractions against *M. smegmatis* and *Escherichia coli* biofilms. Healthcare facilities are a well-known high-risk environment for the transmission of *M. tuberculosis* [53]. The results of this study suggest that it is essential to stop biofilm formation at an early stage before adhered mycobacterial cells develop mature biomass. This may infer the need for consistent cleaning practices in the hospital setting for high-touch or high-contact surfaces, which may involve routine inspections.

### 2.8. LC-MS Assessment of Extracts

**Figure 5:**
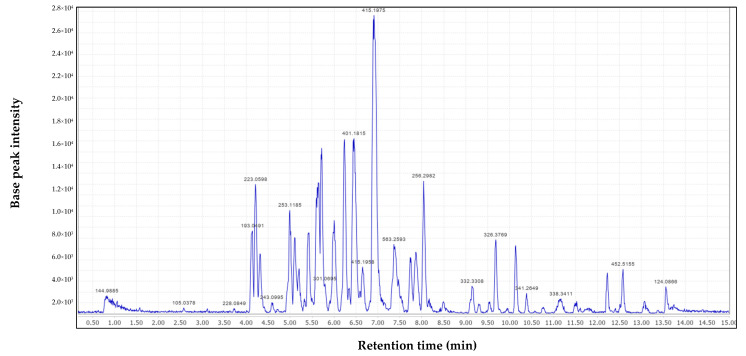
Liquid chromatography–mass spectroscopy chromatogram spectrum analysis of compounds in the acetone subfraction.

Numerous studies have determined that the phytochemical composition of the aerial parts of *A. afra* includes sesquiterpenes, coumarin, flavonoids, coumarin glycosides, phenolic acid, alcoholic glucoside, organic acid, and guaianolide [54]. The results (Table 2) obtained from liquid chromatography–mass spectroscopy (LC-MS) analysis revealed around 17 compounds at different retention times from the subfraction (Figure 5). Furthermore, some of the structures obtained contain imidazole, a molecule that has been reported to be used as a precursor to derive bioactive molecules with various antimicrobial activities [55]. Compounds that contain carboxylic acids in their structure have also been associated with antimicrobial activity [56]. The subfraction contains two compounds with carboxylic acids, namely, 3-[5-(1-aminocyclopropyl)-1,3,4-oxadiazol-2-yl]prop-2-ynoic acid (t: 4.14) and (3S)-4-[[(1S)-2-amino-1-benzyl-2-oxo-ethyl]amino]-3-[[(2S)-2-[[(2S)-2-(3-carboxypropanoylamino)propanoyl]amino]-4-methyl-pentanoyl]amino]-4-oxo-butanoic acid (t: 7.63). Several carboxylic acids and their relative esters have been reported to have antimycobacterial activity against *M. tuberculosis* [57]. The biological activities of the *A. afra* may be attributed to synergistic interactions of these compounds in the subfraction.

### 2.9. Molecular Docking

Molecular docking was used to predict the mechanisms of action of the LC-MS-identified compounds. The two receptors utilized for the study were RNA polymerase binding protein (RbpA)-sigma D, which regulates transcription in mycobacteria [58], and beta-ketoacyl synthase, which helps to synthesize mycolic acids for the mycobacteria cell wall [59]. The four structural domains that makeup RbpA are the sigma interaction domain (SID), the core domain (CD), the basic linker (BL), and the N-terminal tail (NTT) [60,61,62]. Selected receptors are frequently used as potential drug targets in the search for antimycobacterial agents.

The sigma domain was chosen as a receptor in this study because it is critical for the interaction of RbpA with the holoenzyme of RNA polymerase [58]. The molecular interactions of the ligands docked with the two receptors revealed that most of the ligands had higher binding affinities ranging between −4.9 and −7.1 kcal/mol for beta-ketoacyl synthase as compared to RNA polymerase binding protein receptor, which had high binding energies ranging between −4.0 and −6.3 kcal/mol (Table 3). There is better binding energy between the ligands and the enzyme or protein with higher negative values [63].

**Figure 6:**
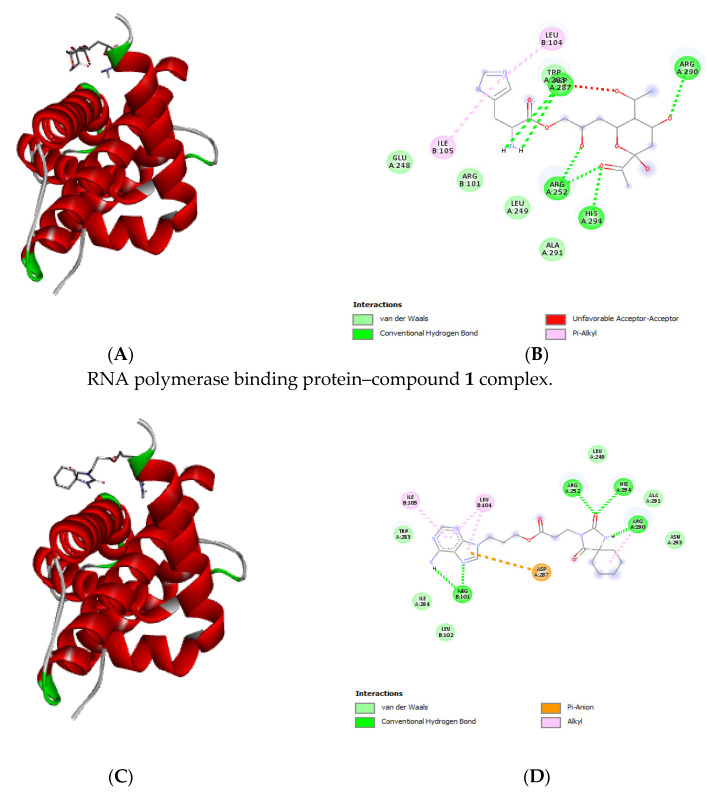
Three-dimensional (**A**,**C**) and two-dimensional (**B**,**D**) structures of compound **1** (3-(6-aminopurin-9-yl)propyl 3-(2,4-dioxo-1,3-diazaspiro[4.5]decan-3-yl)propanoate) and compound **2** ([(2S)-3-[6-acetyl-4,6-dihydroxy-3-[(1R)-1-hydroxyethyl]tetrahydropyran-2-yl]-2-hydroxy-propyl](2R)-2-amino-3-(1H-imidazol-5-yl)propanoate) docked against RNA polymerase binding protein (RbpA).

**Figure 7:**
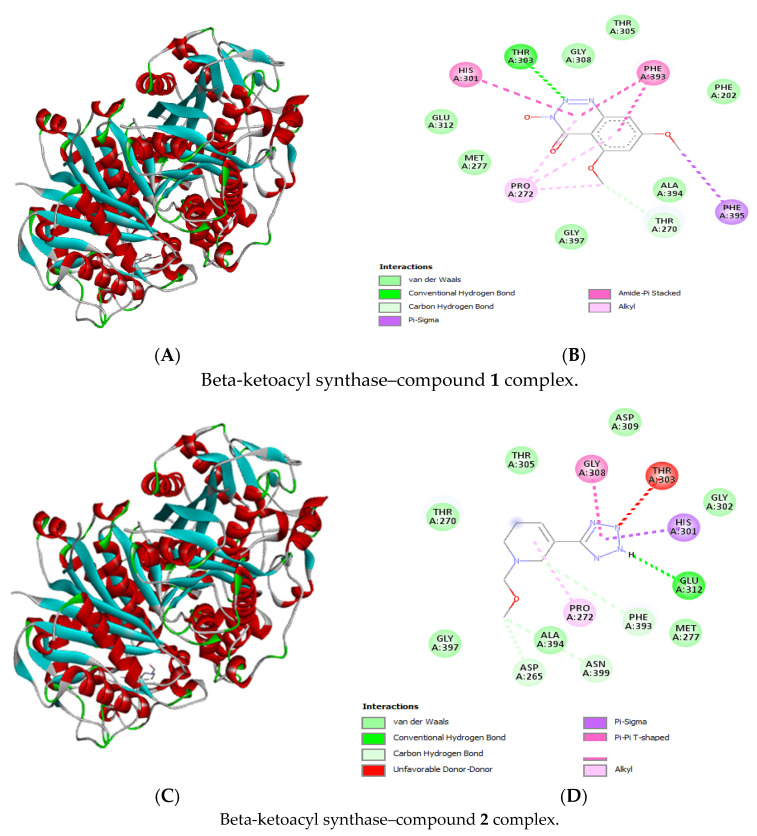
Three-dimensional (**A**,**C**) and two-dimensional (**B**,**D**) structures of compound **1** (3-hydroxy-5,7-dimethoxy-1,2,3-benzotriazin-4-one) and compound **2** (1-(methoxymethyl)-5-(2H-tetrazol-5-yl)-3,6-dihydro-2H-pyridine) docked against beta-ketoacyl synthase.

The bonding interactions (i.e., hydrogen bonds, van der Waals interactions) and amino acids involved are shown in Figure 6 and Figure 7 for complexes docked with RNA polymerase binding protein (RbpA)-sigma D and those docked with beta-ketoacyl synthase. Rifampicin is a well-known broad-spectrum drug [64] and a first-line TB drug inhibiting transcription by targeting RNA polymerase [65]. *M. smegmatis* is used as a surrogate for in vitro evaluations of TB drug discovery, and it possesses cellular structure and metabolic properties, like those of the actual pathogen *M. tuberculosis*; for this reason, it can be inferred that the different mechanisms observed from the docking results apply to both organisms. The two receptors utilized in the study are also present in both species. Therefore, evaluating the effects of treatment combinations involving subfraction and rifampicin may lead to the development of medication that inhibits *M. tuberculosis* growth.

## 3. Materials and Methods

### 3.1. Plant Collection

The aerial parts of the *A. afra* plant were collected during autumn in Haenertsburg (Limpopo), South Africa. The verification of the plant species was conducted by Dr. E Bronwyn at the Larry Leach Herbarium at the University of Limpopo (Voucher number: SS223). The plant material was dried at room temperature and ground to a fine powder.

### 3.2. Plant Extraction

One gram of the plant material was extracted with 10 mL of acetone. Each mixture was placed in a shaking incubator (Corning^®^ LSE^™^) (Merck Life Science (Pty) Ltd., Modderfontein, South Africa) for 30 min at 200 rpm, and the procedure was repeated twice for 20 min each to allow for exhaustive extraction of the plant material. The crude extracts were filtered through Whatman no. 1 filter paper and evaporated under a stream of cold air at room temperature.

### 3.3. Phytochemical Screening

The total phenolic, flavonoid, and tannin contents were quantified using the colorimetric methods described by Tambe and Bhambar [66]. Tannic acid (Merck Life Science (Pty) Ltd., Modderfontein, South Africa) was used to standardize the total phenolics, quercetin was used for the flavonoids, and gallic acid (Merck Life Science (Pty) Ltd., Modderfontein, South Africa) was used to quantify the total tannin contents.

### 3.4. Antioxidant Activity of Acetone Extract

The free radical scavenging activity was quantified using 2,2-diphenyl-1-picrylhydrazyl (DPPH) (Merck Life Science (Pty) Ltd., Modderfontein, South Africa) [67]. Briefly, 2 mL of 0.2 mmol/L of DPPH solution dissolved in methanol was mixed with an equal volume of the extracts (15.63–250, μg/mL). The mixtures were vortexed and incubated in the dark for 30 min. The untreated DPPH solution was used as a control. Ascorbic acid served as the standard. The solutions were analyzed with a UV/VIS spectrophotometer (Genesys 10S UV-VIS) (Thermo Scientific, Johannesburg, South Africa) at a wavelength of 517 nm. The percentage inhibition was calculated using the following formula:

% Percentage inhibition = (ODc − ODt)/(ODc) × 100(1)

Key: ODc = control solution, ODt = absorbance of treatment

### 3.5. Antimycobacterial Screening

#### 3.5.1. Bacterial Culture and Maintenance

*Mycobacterium smegmatis* (ATCC 1441) was obtained from Professor Green in the Department of Biotechnology and Food Technology, University of Johannesburg. Middlebrook 7H10 agar (Merck Life Science (Pty) Ltd., Modderfontein, South Africa) plates supplemented with oleic albumin dextrose catalase (OADC) (complete media) (Becton Dickinson (Pty) Ltd., Johannesburg, South Africa) were used to maintain *M. smegmatis* cultures at 4 °C. A colony was inoculated in complete media and incubated at 37 °C for 24 h for bioassays.

#### 3.5.2. Bioautography Assay

Qualitative analysis of potential antimycobacterial phytochemicals was carried out using the bioautography method described by Beque and Kline [68]. Ten milligrams per milliliter (10 mg/mL) of the extract was used to spot thin-layer chromatography plates, which were then developed using combinations of mobile phases, which were representative of polarities that ranged from non-polar to polar. The following compositions of the mobile phases were prepared: non-polar: benzene/ethanol/ammonium hydroxide (90:10:1) (BEA) and intermediate chloroform/ethyl acetate/formic acid (25:20:5) (CEF); polar: ethyl acetate/methanol/water (80:11:10) (EMW). The plates were then dried at room temperature, and once dried, an overnight *M. smegmatis* culture (OD600, 0.9) was sprayed onto the dried TLC plates at biosafety level 2. The plates were incubated overnight at 37 °C in 100% humidity. Following a spray application of a 2 mg/mL *p*-iodonitrotetrazolium chloride (INT) solution (Merck Life Science (Pty) Ltd., Modderfontein, South Africa), the plates were incubated for 3 h at 37 °C. The zones indicated the inhibition of bacterial growth.

#### 3.5.3. Broth Microdilution Assay

The minimum inhibitory concentrations (MICs) of the crude extract and fractions were quantified using the assay described by Eloff [69]. The culture was inoculated in Middlebrook 7H9 broth at a starting OD600 0.1. The culture was incubated at 37 °C at 150 rpm and allowed to grow to an OD600 0.8–0.9 to maintain the cells in the exponential phase. In 96-well microtiter plates, 100 μL of the extracts (0.02–2.5 mg/mL) were prepared, and this was followed by 100 μL of the prepared culture. The positive control was rifampicin. The negative controls consisted of 25% acetone in the medium and the medium alone. The plates were incubated at 37 °C for 24 h. Subsequently, 40 μL of 0.2 mg/mL INT was added to the microtiter plate wells, and then the plates were incubated for an additional 30 min at 37 °C. The clear wells were indicative of growth inhibition.

#### 3.5.4. Bioassay-Guided Fractionation Using Column Chromatography

Silica gel column chromatography was used to fractionate active compounds by bioassay-guided fractionation, as reported by Kotze and Eloff [70]. The crude acetone extracts obtained from serial exhaustive extraction were fractionated with open-column chromatography. The extracts (12.789 g) were mixed with silica gel to a paste-like consistency and loaded onto the column. The column (height: 35 cm; radius: 3 cm) was filled with silica gel 60 (particle sizes of 0.063–0.200 mm). The extracts were eluted using 1.2 L of different solvent systems (i.e., hexane/ethyl acetate and ethyl acetate/methanol). The concentrated fractions were collected until each 1.2 L of eluent solvents was finished, and a rotary evaporator was used to reduce the final volume to 100 mL. Thereafter, the solvents were left to evaporate at ambient temperature, and the mass of fractions was quantified.

#### 3.5.5. Growth Curve of *M. smegmatis* After Extract Treatment

The growth kinetics *of M. smegmatis* were evaluated during 24 h of growth in the presence of extracts of *A. afra* [71]. *M. smegmatis* culture grown overnight was inoculated into conical flasks containing 20 mL of Middlebrooks 7H9 supplement broth to start OD600 0.1. Subsequently, it was treated with the extracts (0.5×MIC, MIC, 2×MIC, and 4×MIC mg/mL) and incubated in a shaker incubator (Corning^®^ LSE^™^) (Merck Life Science (Pty) Ltd., Modderfontein, South Africa) at 37 °C. Growth was followed by monitoring the OD600 at intervals of 3, 6, 9, 18, and 24 h. The untreated culture was the positive control of the experiment, and the uninoculated medium was the negative control.

### 3.6. Evaluation of Plant Extract Cytotoxicity

#### 3.6.1. Cell Culture and Maintenance

THP monocytes (Thermo Scientific, Johannesburg, South Africa) were cultured in RPMI-1640-glutamine medium (Inqaba biotec, Tshwane, South Africa). Then, 10% fetal bovine serum (FBS) (Inqaba biotec, Tshwane, South Africa) was used to supplement the medium in T75 flasks (Inqaba biotec, Tshwane, South Africa). The cells were incubated at 37 °C and CO_2_ (5%). Every 2–3 days, the cells were passaged and kept at a density of 2–6 × 10^5^ cells/mL.

#### 3.6.2. Differentiation Induction

The THP-1 monocytes were centrifuged in 50 mL sterile tubes at 1000 rpm for 10 min at 4 °C. The pelleted cells were reconstituted in a complete RPMI-1640 supplemented with 100 ng/mL of phorbol 12-myristate 13-acetate (PMA) (Inqaba biotec, Tshwane, South Africa). The resuspended culture (200 µL) was seeded in 96-well plates at a final cell concentration of 100,000 cells/well. The cells were incubated for 24 h in a humidified incubator at 37 °C and 5% CO_2_ to allow differentiation. The spent medium was removed from the wells, and the cells were washed twice using a pre-warmed RPMI-1640 medium [72].

#### 3.6.3. Cytotoxicity Test

The extracts were dissolved in 100% DMSO (Merck Life Science (Pty) Ltd., Modderfontein, South Africa) to obtain 100 mg/mL working stock concentrations. The growth medium was removed from the wells of adhered naïve macrophages. Extracts (200 µL) that had been diluted in the complete RPMI medium at the relevant concentrations (125–1000 µg/mL) were added to wells. The control wells contained 200 µL of 1% DMSO in complete RPMI, with some wells containing cells while others were left empty (media only), and they were incubated for 24 h at 37 °C with 5% CO_2_. After incubation, the medium was removed from the wells, and 100 mL of complete RPMI medium was added and incubated for 10 min at 37 °C and 5% CO_2_ to remove the cells. The cell suspensions (20 µL) were diluted with equal volumes of trypan blue solution (Merck Life Science (Pty) Ltd., Modderfontein, South Africa), thoroughly mixed, and placed on a hemacytometer and a cover slip. The hemacytometer (Merck Life Science (Pty) Ltd., Modderfontein, South Africa) was placed under a microscope and focused on the cells using a 40X magnification objective lens. The cells were counted using squares of the grid. The cells in each square were counted separately and averaged [73].

### 3.7. Screening of Antibiofilm Activity

#### 3.7.1. Cell Attachment Inhibition

To investigate the anti-adherence potential of the extracts, 100 μL of *M. smegmatis* culture at OD600 0.02 (1.0 × 10^7^ CFU/mL) was added to flat-bottomed 96-well microtiter plates in three replicates and incubated at 37 °C for 4 h without shaking. Subsequently, 100 μL of the extracts were added to make final concentrations that yielded 0.5×MIC, MIC, 2×MIC, and 4×MIC mg/mL. The plates were incubated further at 37 °C for 24 h without shaking [74]. The untreated culture served as the positive control, 25% acetone in media and sterile media served as the negative controls, and rifampicin (Inqaba biotec, Tshwane, South Africa) was used as the standard for the assay.

#### 3.7.2. Inhibition of the Preformed Biofilm

The antibiofilm activities of the extracts were evaluated by assessing their potential to eradicate preformed biofilm biomass [74]. An aliquot of 100 μL of *M. smegmatis* cultures with OD600 0.02 was added to 96-well microtiter plates and incubated at 37 °C for 24 h (early biofilm) and 48 h (mature biofilm) without shaking. After each incubation, the biofilms were treated with 100 μL of the extracts with final concentrations of 0.5×MIC, MIC, 2×MIC, and 4×MIC mg/mL in fresh media. The microtiter plates were then incubated for another 24 h. Untreated microbial cells served as a positive control, and 25% acetone in media, with some wells containing cells while others were left empty (media only), served as negative controls. The biomass was quantified using a modified crystal violet (Inqaba biotec, Tshwane, South Africa) staining method [75].

#### 3.7.3. Crystal Violet Staining Assay

A crystal violet assay was used to quantify the inhibition of cell attachment and eradication of pre-formed biofilms [75]. After the respective treatments, the plates were washed three times with sterile distilled water and heated in an oven to dry at 60 °C for 45 min. The attached biomass was stained for 15 min with 100 μL 0.1% crystal violet. After staining, the plates were washed three times and air-dried. In the dried plates, 125 μL of methanol was added to dissolve the absorbed stain. The solution (100 μL) was added to 96-well round bottom plates, and the absorbance of the solutions was read at 590 nm. The percentage inhibitions were estimated using the following equation:

Percentage inhibition = (ODcontrol − ODtreatment)/(ODcontrol) × 100(2)

### 3.8. LC-MS Analysis

LC-MS analysis was performed using a Waters Synapt G2 qTOF mass spectrometer (Waters Corporation, Milford, MA, USA). Before analysis, fraction extracts were centrifuged for 10 min at 12,000 rpm. The phytoconstituents were separated using a HSS T3 column from water, 2.1 × 150 mm. Two mobile phases (A) and (B) were used, where (A) consisted of 0.1% formic acid (Inqaba biotec, Tshwane, South Africa) in water and (B) had 5 mM ammonium formate acetonitrile (Inqaba biotec, Tshwane, South Africa). A 5 µL volume of extracts was injected into the analytical column for analysis. The sample flow rate was set at 0.4 mL/min. Mass fragmentations were identified using a spectrum database for organic compounds [76].

### 3.9. In Silico Studies

#### 3.9.1. Preparation of Protein Receptors and Ligands

The target receptors of the RNA polymerase binding protein (RbpA)-sigma D (PDB ID: 4X8K) and beta-ketoacyl synthase (PDB ID: 4EWG) were procured from the Protein Data Bank (PDB) (www.rcsb.org) (accessed on 25 June 2024) (Piscataway, NJ, USA), and the three-dimensional (3D) structures of the LC-MS-identified ligands were downloaded from the PubChem compound database of the National Centre for Biotechnology Information (NCBI). The protein receptors were refined to improve the efficiency of docking by eliminating heteroatoms, water molecules, and other ligand groups before adding polar hydrogens using Discovery Studio software version 4.1 (2021 client) [77].

#### 3.9.2. Molecular Docking

Molecular docking was performed using AutoDock Vina version 1.5.6. The binding sphere for 4X8K (32.746800, 41.522800, and 23.813600 for x, y, and z centers, respectively) and 4EWG (26.985758, 21.606515, and 85.843121 for x, y, and z centers, respectively) were identified from the active sites of the receptors using the Discovery Studio 4.1. Binding affinities were estimated by evaluating the binding scores of the ligand–receptor complexes using AutoDock Vina version 1.5.6 [78]. The lowest binding energy scores of the ligand–receptor complexes were selected as the best-docked structures. Subsequently, the docked complexes were imported to Discovery Studio version 4.1 to visualize the generated 2- and 3-dimensional (2D and 3D) structures [77].

### 3.10. Statistical Analysis

Two-way analysis of variance (ANOVA) was used for statistical comparisons using GraphPad Prism version 9.0. Differences were considered significant when *p* < 0.05; in contrast, the non-significance values, *p* < 0.05, were indicated.

## 4. Conclusions

This study confirmed the antioxidant, antibiofilm, and antimycobacterial properties of the medicinal plant *A. afra*. These biological activities are linked to the presence of various phenolic compounds, including phenolic acids, flavonoids, and tannins, which exhibited significant antioxidant effects in the acetone crude extract. The most effective subfraction demonstrated bacteriostatic antimycobacterial activity, inhibiting the growth of planktonic *M. smegmatis* for approximately 9 h. In silico analyses indicated that this subfraction might inhibit mycobacterial growth by targeting RNA polymerase binding proteins and beta-ketoacyl synthases. Further studies may be required to investigate the potential synergistic effects between the *A. afra* subfractions and antibiotics, such as rifampicin, that target the mycobacterial cell wall synthesis. Antibiofilm analysis indicated that the cell adherence phase was the most vulnerable stage in the biofilm development of *M. smegmatis*. While early-stage biofilms were sensitive to the subfraction, mature biofilms exhibited a higher resistance to eradication. To better identify the targets related to inhibition, it is essential to concentrate on the initial phases of mycobacterial biofilm formation.

## Figures and Tables

**Table 1:** Total polyphenolic content, antimycobacterial activity, and cytotoxicity of *A. afra* extracts.

	Polyphenolic Content		
Sample	TPC (mg TAE/g)	TFC (mg QE/g)	TTC (mg GAE/g)
Crude Extract	599.7 ± 3.5	60.6 ± 1.3	87.2 ± 4.1
	Antimycobacterial activity (MIC mg/mL)		
Crude	0.52 ± 0.22		
F_C1_	0.90 ± 0.10		
F_C2_	0.078 ± 0		
Rifampicin	0.0016 ± 0		
	Cytotoxicity (LC_50_ (μg/mL))		
Crude	172.7		
F_C2_	172.7		
Rifampicin	320.30		

TPC: total phenolic content; TFC: total flavonoid content; TTC: total tannin content; mg TAE/g: milligram tannic acid equivalence/gram of extract; mg QE/g: milligram quercetin equivalence/gram of extract; mg GAE/g: milligram gallic acid equivalent/gram of extract; MIC: minimum inhibitory concentration; LC_50_: 50% lethal concentration; F_C1_: 1st column fraction; F_C2_: 2nd column fraction.

**Table 2:** LC-MS analysis of *A. afra* acetone subfraction (F_C2_).

Isotopic Mass	Formula	Identifier	Retention Time	Name	Database
145.0	C_2_HN_4_O_4_	87396588	0.83	4-nitro-3-oxo-4H-triazol-3-ium-5-one	PubChem
144.989	C_3_HN_2_O_5_	88813764	3.12	1-oxido-3-oxo-imidazolidine-1,3-diium-2,4,5-trione	PubChem
228.085	C_7_H_16_O_8_	89009828	3.73	3,4,5-trihydroperoxy-1-methoxy-hexan-2-ol	PubChem
193.049	C_8_H_7_N_3_O_3_	82669819	4.14	3-[5-(1-aminocyclopropyl)-1,3,4-oxadiazol-2-yl]prop-2-ynoic acid	PubChem
223.059	C_9_H_9_N_3_O_4_	70341629	4.21	3-hydroxy-5,7-dimethoxy-1,2,3-benzotriazin-4-one	PubChem
253.117	C_10_H_15_N_5_O_3_	23487968	4.99	N4-allyl-N6-(2-methoxyethyl)-5-nitro-pyrimidine-4,6-diamine	PubChem
401.18	C_17_H_27_N_3_O_8_	91595513	6.46	[(2R)-2-[(1S)-1-[(2S)-2,6-diaminohexanoyl]oxy-2-hydroxy-ethyl]-4,5-dioxo-tetrahydrofuran-3-yl] (2S)-pyrrolidine-2-carboxylate	PubChem
415.195	C_18_H_29_N_3_O_8_	89564021	6.65	[(2S)-3-[6-acetyl-4,6-dihydroxy-3-[(1R)-1-hydroxyethyl]tetrahydropyran-2-yl]-2-hydroxy-propyl] (2R)-2-amino-3-(1H-imidazol-5-yl)propanoate	PubChem
415.197	C_19_H_25_N_7_O_4_	56284089	6.91	3-(6-aminopurin-9-yl)propyl 3-(2,4-dioxo-1,3-diazaspiro[4.5]decan-3-yl)propanoate	PubChem
195.112	C_8_H_13_N_5_O	82365250	6.89	1-(methoxymethyl)-5-(2H-tetrazol-5-yl)-3,6-dihydro-2H-pyridine	PubChem
195.112	C_8_H_13_N_5_O	69463083	6.91	1-ethoxy-2-methyl-pyrrolo[1,2-b][1,2,4]triazole-5,7-diamine	PubChem
563.259	C_26_H_37_N_5_O_9_	101614490	7.63	(3S)-4-[[(1S)-2-amino-1-benzyl-2-oxo-ethyl]amino]-3-[[(2S)-2-[[(2S)-2-(3-carboxypropanoylamino)propanoyl]amino]-4-methyl-pentanoyl]amino]-4-oxo-butanoic acid	PubChem
338.341	C_20_H_42_N_4_	71442591	11.17	2-hexadecyl-3,5-dimethyl-3H-1,2,4-triazol-4-amine	PubChem

**Table 3:** Binding energies of compounds detected from the acetone fraction of *A. afra* docked against receptors of *M. smegmatis*.

Target	Protein Data Bank Code	Name of Ligands	Binding Energies (kcal/mol)
RNA polymerase binding protein (RbpA)	4X8K	[(2S)-3-[6-acetyl-4,6-dihydroxy-3-[(1R)-1-hydroxyethyl]tetrahydropyran-2-yl]-2-hydroxy-propyl](2R)-2-amino-3-(1H-imidazol-5-yl)propanoate	−5.4
		3-(6-aminopurin-9-yl)propyl 3-(2,4-dioxo-1,3-diazaspiro[4.5]decan-3-yl)propanoate	−6.3
Beta-ketoacyl synthase	4EWG	3-hydroxy-5,7-dimethoxy-1,2,3-benzotriazin-4-one	−7.1
		1-(methoxymethyl)-5-(2H-tetrazol-5-yl)-3,6-dihydro-2H-pyridine	−7.1

## Data Availability

The data used to support the findings of this study are available in the article.
